# Structured assessment of a cadaveric orthopedic surgical training program of small animal surgeons in training: A prospective observational pilot study

**DOI:** 10.1111/vsu.70033

**Published:** 2025-10-31

**Authors:** Dominique Leu, Antonio Pozzi, Lucas A. Smolders, Brian Park, Heidi Radke

**Affiliations:** ^1^ Department of Clinical Veterinary Medicine, Division of Surgery, Vetsuisse Faculty University of Zürich Zürich Switzerland

## Abstract

**Objective:**

To determine whether structured and supervised cadaveric surgical training improves technical skills in expert and self‐assessments and enhances self‐perception of surgical trainees.

**Study design:**

Prospective observational study.

**Sample population:**

Seven small animal ECVS residents and surgical interns.

**Methods:**

A cadaveric orthopedic training program, consisting of six supervised training sessions, weekly self‐directed training, and one one‐to‐one training session with direct feedback, was conducted over 3 months. Each participant performed a set of surgical procedures on feline cadavers before and after the training period. The procedures were videotaped and subsequently evaluated by three board‐certified surgeons (expert assessment [EA]) and the participants themselves (self‐assessment [SA]) using the objective structured assessment of technical skills (OSATS) global rating scale (GRS). OSATS GRS scores were analyzed using the Wilcoxon signed‐rank test, Kruskal‐Wallis test, and Cohen's kappa coefficient (Κ).

**Results:**

Comparing pre‐ and post‐training assessments, median EA scores increased from 17/35 to 23/35 (*p* = .001). Median SA scores increased from 17/35 to 25/35 (*p* = .018). There was slight to substantial inter‐assessor agreement (Κ = 0.04–0.65) and poor agreement between SA and EA (Κ = 0).

**Conclusion:**

Trainee surgeons improved their technical skills in expert and self‐assessments. However, the interassessor agreement was strong for only two of the three assessors. Despite limitations, the OSATS GRS enhanced the objectivity of technical surgical skills assessment.

**Clinical significance:**

This study represents the first step in devising a meaningful training outside the operating room for veterinary surgical residents. The OSATS GRS as an assessment tool warrants further investigation.

## 1 INTRODUCTION

Residents in small animal surgery undergo a rigorous training program supervised by veterinary surgical specialists to become a diplomate of a recognized specialty college.

An increasing demand for greater operating room efficiency, together with stricter limitations on residents' working hours and reduced operating room exposure, presents significant challenges in the education of trainee surgeons in both human and veterinary surgery.^1^, ^2^, ^3^, ^4^ Residency training programs often rely on subjective verbal feedback provided directly in the operating room without a system to document intraoperative assessment and progress.^1^, ^5^, ^6^ Nevertheless, regular structured feedback has an important influence on the adult learning process and enhances trainees' self‐perception of technical surgical skills.^7^, ^8^, ^9^, ^10^ An accurate self‐perception of medical competence is characterized by a strong correlation between an individual's self‐assessment and external objective evaluations.^11^, ^12^ A good self‐perception is an essential form of quality assurance, as it contributes to improved patient safety in the operating room and thus represents a key aspect of surgical specialization.^13^, ^14^ Adding additional training opportunities to the curriculum may help overcome these challenges and improve both, the practical skills and self‐perception of the trainee surgeons.

In human surgical training programs, the impact of practical training sessions outside the operating room has been investigated using different scoring systems. The most commonly used scoring system is the objective structured assessment of technical skills (OSATS) score, demonstrating a high inter‐rater reliability and construct validity.^5^, ^9^, ^15^, ^16^ It was initially designed as a combination of a task‐specific checklist, a global rating scale, and a pass‐fail assessment.^16^, ^17^ The task‐specific checklist deconstructs a task into multiple steps using a binary scoring system.^15^, ^18^ In contrast, the global rating scale evaluates the general quality of the performance in predefined categories using a Likert scale.^15^, ^18^ The pass‐fail assessment documents adverse events.^19^ The combined approach has the disadvantage of being time‐consuming and procedure‐specific.^15^, ^18^ Consequently, several authors have reported the OSATS global rating scale (OSATS GRS) as the sole assessment tool in their studies, with good reliability and validity.^10^, ^13^, ^15^, ^20^

In the veterinary literature, there is currently no data evaluating the effect of a structured veterinary surgical training program with supervised and unsupervised training sessions on cadavers during a registered surgical specialist training in small animal surgery. Furthermore, there is no recommendation from specialty colleges (such as the European or American College of Veterinary Surgeons [ECVS or ACVS]) for structured, regular, and supervised training in technical surgical skills. To close this gap, this study aimed to determine whether structured and supervised cadaveric surgical training improves technical skills in expert and self‐assessments using the OSATS GRS and enhances the self‐perception of surgical trainees. We hypothesized that structured and supervised orthopedic cadaveric surgical training would lead to improvements in technical surgical skills, as assessed by both expert and self‐assessments. We also hypothesized that the training would lead to an improved self‐perception of the participants throughout the training.

## 2 MATERIALS AND METHODS

All first and second‐year small animal ECVS residents and all surgical interns (veterinarians on a 1‐year program preparing for a surgical residency) at the Small Animal Clinic, University of Zurich, were invited to participate in a 3‐month surgical training program on cadavers focusing on basic orthopedic skills. Participation was voluntary, and no incentive was provided. Three ECVS/ACVS diplomates acted as training instructors and evaluated the outcome as assessors. Participants were informed that they would receive formative feedback and that the results would be scientifically assessed without affecting any of their educational or clinical evaluations. Ethics approval for this study was obtained in April 2023 from the CEBES Review Board, the Ethics Committee of the Institute of Bioethics and the History of Medicine, University of Zurich (28042023). All cat cadavers were client‐owned and euthanized for reasons unrelated to the study. All clients provided written consent to use the cadavers for research purposes.

### 2.1 Training

The cadaver training consisted of the following elements: (1) Six instructor‐guided practical training sessions (1.5–2 h each), (2) four hours of self‐directed training per week, and (3) a 1–1.5‐h one‐to‐one training session with direct feedback from one of the instructors. In each instructor‐guided training session, specific surgical training elements (awareness of anatomy, basic surgical skills, advanced surgical skills, problem‐solving, movement efficiency), as well as specific surgical procedures, were introduced and practiced under supervision. The following topics were covered:

1. Sharp and blunt dissection and instrument handling.
2. Tissue handling, suturing and hand tie.
3. Approaches to long bones (humerus, radius, femur, tibia) and pelvis.
4. Approaches to joints (elbow‐, carpal‐, coxofemoral‐, stifle‐, and tarsal joint).
5. Retraction, surgical exposure and efficiency.
6. Specific procedures (application of a lag screw or positional screw; use of cerclages such as twist‐knot, single‐loop and double loop; osteotomy of the greater trochanter of the femur and concurrent fixation with cerclage compression wiring; placement of an intramedullary pin).

Participants received a study list of procedures and approaches to be assessed on by the end of the training period (Table S1). The list contained general surgical principles, a selection of the most common approaches to long bones and joints, and specific procedures that allowed for the training and the assessment of multiple surgical skills within a single procedure. Participants kept a case log to register time and procedures. The group‐guided training sessions, individual one‐to‐one sessions, self‐guided training sessions, and relevant literature studies all contributed to the case log.

### 2.2 Assessment

To evaluate the effect of the overall training, each participant was recorded on camera while performing three specific procedures on a cat cadaver (without assistance) before (pretraining assessment [preTrngA]) and after the training period (post‐training assessment [postTrngA]). Participants wore sterile gloves and a surgical gown to cover their hands and arms. Recordings were soundless, and the order of participants was randomized between preTrngA and postTrngA. One video camera was positioned next to the trainee and recorded the surgical field. It captured only the cadaveric specimen, the hand and arm movements of the participants, and the instruments used to perform the procedure. The participants were unaware of which procedures from the training catalog they would perform during the recording. Without the knowledge of the participants, the first and third procedures were predefined and identical for all participants to facilitate comparison. The second procedure was randomly assigned by drawing lots. The following procedures were recorded:

1. Approach to the tibia, creating a long oblique fracture with an oscillating saw, placement of a lag screw, a positional screw, and a twist‐knot cerclage around the long oblique fracture.
2. Approach to a joint, randomly assigned (elbow‐, carpal‐, hip‐, stifle‐, or tarsal‐joint).
3. Dorsal approach to the coxofemoral joint with a greater trochanteric osteotomy and subsequent fixation with cerclage compression wiring.

Each instructor assessed each participant based on the video recordings (expert assessment [EA]). Each participant self‐assessed their performance directly after completing the procedures (self‐assessment [SA]). The participants did not receive feedback from the instructors on their video performance in preTrngA. Experts and participants both used the OSATS GRS (Table S2).^16^, ^17^ The OSATS GRS category “use of assistants” was modified to “use of proper retraction” as no assistants were used.

### 2.3 Data analysis and statistics

The results of SA and EA were analyzed and compared. The data were statistically evaluated using SPSS software (version 27.0, IBM Corp., Armonik, New York). A Wilcoxon signed‐rank test was used to compare the OSATS scores before and after the training program for both EA and SA. A Kruskal‐Wallis test was used to assess the overall difference between EA and SA. A *p*‐value < .05 was considered statistically significant. To judge the self‐perception of surgical trainees, the agreement of OSATS GRS scores between individual EA and SA scores was evaluated using the weighted Cohen's kappa coefficient (Table 1).^21^

**TABLE 1:** Interpretation of Cohen's kappa coefficient.

Kappa	Agreement		
<0	Less than chance agreement/poor agreement		
0.01–0.20	Slight agreement		
0.21–0.40	Fair agreement		
0.41–0.60	Moderate agreement		
0.61–0.80	Substantial agreement		
0.81–0.99	Almost perfect agreement		

## 3 RESULTS

Seven veterinary surgeons in training (3 ECVS residents and 4 surgical interns) were enrolled in this study. The residents had more surgical experience (median 3.0 years; range, 2.5–8.0 years) compared to the surgical interns (median 1.3 years; range, 1.0–6.0 years). During the 3‐month training period, the median total time invested in training was 45.8 h (range, 24.5–59.5 h), with a median of 4.0 h per week (range, 2.0–4.5 h).

### 3.1 Expert assessment

**FIGURE 1:**
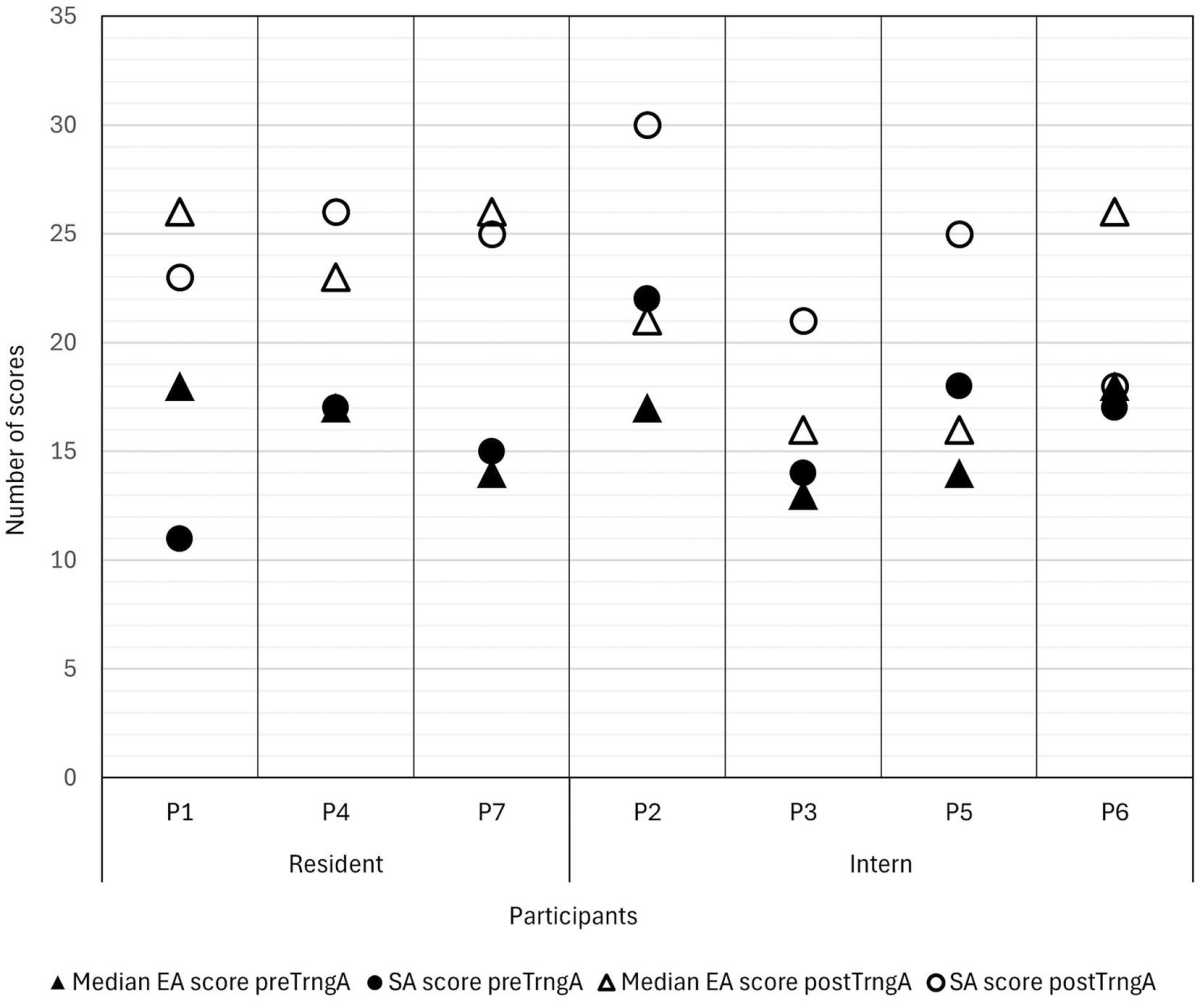
Self‐assessment (SA) compared to expert assessment (EA) in pretraining assessment (preTrngA) and post‐training assessment (postTrngA). Median EA score preTrngA = median score from all three expert assessments in pretraining assessment; SA score preTrngA = score from each self‐assessment in pretraining assessment; Median EA score postTrngA = median score from all three expert assessments in post‐training assessment; SA score postTrngA = score from each self‐assessment in post‐training assessment; P1–P7 = participants 1–7.

All experts were ECVS or ACVS diplomates with variable amounts of clinical experience as diplomate surgeons (expert 1: 18 years, expert 2: 4 years, expert 3: 20 years). An improvement of the OSATS GRS scores from preTrngA to postTrngA was noted based on EA (*p* = .001). Median scores for assessor 1 at preTrngA were 17/35 (range, 14–21 scores) and 23/35 (range, 16–28 scores) at postTrngA, respectively (Figure 1). Similar results were observed for assessor 2, with a median score at preTrngA of 16/35 (range, 13–18 scores) and 23/35 (range, 15–27 scores) at postTrngA. Lower overall scores were given by assessor 3. However, an improvement was still observed, with median scores at preTrngA of 15/35 (range, 10–18 scores) increasing to 17/35 (range, 14–21 scores) at postTrngA. We observed a moderate (Κ = 0.54) to substantial (Κ = 0.65) interassessor agreement between two of three assessors comparing preTrngA and postTrngA, respectively (Table 2). There was only slight to fair agreement comparing the evaluation of the third assessor (Κ = 0.04–0.28).

**TABLE 2:** PreTrngA and postTrngA agreement analysis.

	PreTrngA		PostTrngA	
	kappa	Agreement	kappa	Agreement
Expert 1‐Expert 2	0.54	Moderate	0.65	Substantial
Expert 1‐Expert 3	0.28	Fair	0.04	Slight
Expert 2‐Expert 3	0.06	Slight	0.16	Slight
Expert 1‐Participants	0	Poor	0	Poor
Expert 2‐Participants	0.18	Slight	0	Poor
Expert 3‐Participants	0	Poor	0.07	Slight

Abbreviations: PostTrngA, post‐training assessment; PreTrngA, pretraining assessment.

### 3.2 Self‐assessment

SA revealed an improvement (*p* = .018) in OSATS GRS scores comparing preTrngA (median scores 17/35; range, 11–22 scores) to postTrngA (median scores 25/35; range, 18–30 scores). Residents tended to rate themselves lower in preTrngA (median scores 15/35; range, 11–17 scores) compared to surgical interns (median scores 19/35; range, 14–22 scores). In contrast, the residents demonstrated a higher improvement in median scores at postTrngA (25/35; range, 23–26 scores) compared to the surgical interns (23/35; range, 21–30 scores).

### 3.3 Expert assessment compared to self‐assessment

There was only slight (K = 0.18) to poor (K = 0) agreement between EA and SA scores (Table 2). No statistical difference was observed between EA and SA scores (*p* = .12). Trainees tended to overrate or underrate their performance compared to EA in preTrngA and postTrngA (Figure 1). A higher level of agreement was observed for surgical residents (Κ1 = 0.35, Κ2 = 0.4, Κ3 = 0.31) than for surgical interns (Κ1 = 0, Κ2 = 0, Κ3 = 0.1) when compared to each expert individually (expert 1, expert 2 and expert 3).

## 4 DISCUSSION

Based on this pilot study, the basic orthopedic surgical skills of residents and surgical interns improved after undergoing a 3‐month, structured, and supervised training program. The OSATS GRS, which was not previously employed to assess veterinary surgical residents and interns, was used to measure improvements through expert and self‐assessments. The low interassessor agreement suggests that future studies require a strong emphasis on improving the interassessor agreement to validate such a training program as a model.

In contrast to our results, several human studies reported a strong interassessor agreement when utilizing the OSATS GRS to assess surgeons' technical skills.^8^, ^10^, ^13^, ^22^ Whilst in some studies the OSATS GRS was used without specific preparation,^8^, ^10^, ^13^ others performed rater training to agree upon standards for each score level.^20^, ^22^ Our expert assessors had extensive experience in assessing surgical trainees. They reviewed and discussed the OSATS scale prior to the training. Nonetheless, they may still have had a different interpretation of skills expected at the level of a surgical trainee corresponding to each OSATS GRS score. Therefore, further assessor training to standardize expectations of surgical technical skills within each OSATS category could have enhanced the interassessor agreement.^23^, ^24^, ^25^

Additionally, there are some constraints that are inherent to the design of the OSATS GRS: Certain categories in the OSATS GRS are susceptible to a subjective assessment (i.e., instrument handling, respect for tissue).^15^ Furthermore, the uncertainty in judging key elements of a specific procedure, adverse events (i.e., breakage of a screw), and the quality of the final result may also have contributed to the poor inter‐assessor agreement.^1^, ^19^, ^26^ Reznick et al. developed the original OSATS evaluation as a combination of the GRS with a task‐specific checklist and a pass‐fail assessment.^16^ The checklist must be designed and validated separately for each procedure, which is a time‐consuming and burdensome process.^15^, ^18^ Several authors reported an excellent correlation between the results of the checklist and the GRS.^19^, ^27^, ^28^ The pass‐fail assessment was found to be overly subjective in the evaluation and did not improve the overall assessment.^19^, ^27^ Nonetheless, future studies may assess the impact of additions to the GRS or alternative assessment tools to address these limitations.

Progress evaluations of human and veterinary surgical trainees face the same difficulties, with the main challenge being to reliably assess the surgical skills of surgical trainees during their specialist training. Although several studies proposed valid results using the OSATS GRS,^10^, ^13^, ^15^, ^20^ subjectivity in the assessment and insufficient reliability when applying the OSATS GRS were reported in other human medical papers.^4^, ^5^, ^25^, ^29^ In our sample, the OSATS GRS was able to detect a change in skills, as the scores in postTrngA compared to preTrngA improved for all assessors. This result complies with other human studies.^20^, ^22^, ^30^ It suggests that the GRS is suitable for educational purposes, as it can be applied across various procedures without requiring adaptations, enabling the acquisition of timely feedback and providing insights into trainees' learning curve to identify the potential requirements for additional support.^20^, ^30^, ^31^ Due to the inherent limitations (present in human as well as in the veterinary field), its application in high‐stakes summative assessments is, however, not recommended.^5^, ^29^ Further exploration of the OSATS GRS is recommended to assess surgical skills for veterinary surgical trainees over an extended period and in a larger population.

The self‐perception of the surgical trainees in our sample did not improve throughout the training as there was no overall agreement between self‐ and expert assessments in preTrngA and postTrngA. Interestingly, residents demonstrated a better agreement between SA and EA compared to surgical interns when the groups were analyzed separately. This finding is consistent with other reports of a positive correlation between surgical experience and a more realistic self‐assessment.^7^, ^9^, ^12^, ^22^, ^32^, ^33^ Effective self‐assessment is a complex process that requires skills and reflective practice to provide a realistic self‐appraisal.^34^ Three months of intense training are not sufficient to become an experienced surgeon. A longer timeframe with continued expert feedback could thus lead to a more accurate self‐perception of surgical skills.^7^, ^9^

Whilst our results should be interpreted carefully, one of the main findings of this pilot study is that a structured training program performed on cadavers is feasible in a small animal surgery training program. During the study, we learned that all participants were committed to completing the training log. Structured practical training can, therefore, be effectively integrated into a residency training program. This is a key message for institutions such as ACVS and ECVS, as well as for residency program directors.

Further minor limitations must be considered. Participants wore gloves and a gown with only the surgical field visible, and no sound was recorded in the videos for the expert assessments. Nonetheless, a remaining observer bias cannot be completely ruled out. In addition to the training program, the participants also worked clinically and may have gained practical experience beyond the cadaver training. We addressed this confounding variable by assessing a variety of procedures and skills in pre‐ and postTrngA. The number of participants and experts was low, although it was representative of a veterinary surgical training center. The limited sample size increased the risk of a type II error in the statistical results.

In conclusion, this pilot study demonstrated that structured cadaver training improved the basic orthopedic surgical skills of small animal surgical trainees. Integrating structured cadaveric training into the residency curriculum was feasible during this 3‐month trial period. Despite several limitations, this study represents a first step in devising a meaningful and structured training with an objective assessment of technical skills for veterinary surgical residents and interns. We propose an extended evaluation of the reliability of the OSATS GRS for veterinary trainee surgeons by applying it to a larger population over an extended period. Adding an effective option for routine, objective, and timely feedback to the education of trainee surgeons could have a powerful impact on small animal surgical residency programs.

## AUTHOR CONTRIBUTIONS

Leu D, MVetMed: Contributed to the design of the study, compiled all data, performed the statistical analysis, and drafted and revised the manuscript. Pozzi A, DVM, MS, DipECVS, DACVS (Small Animal), DACVSMR: Contributed to the design of the study, introduced general skills and specific procedures to the participants, performed the expert assessment, and provided scientific, in‐line editing of the manuscript. Smolders LA, DVM, PhD, DipECVS: Contributed to the design of the study, introduced general skills and specific procedures to the participants, and performed the expert assessment. Park B, PhD: Contributed to the design of the study, performed the statistical analysis, and interpreted the data. Radke H, MA, DVM, DipECVS, FRCVS: Contributed to the design of the study, introduced general skills and specific procedures to the participants, performed the expert assessment, oversaw data collection, and provided scientific, in‐line editing of the manuscript. All authors provided a critical review of the manuscript and endorse the final version. All authors are aware of their respective contributions and have confidence in the integrity of all contributions.

## FUNDING INFORMATION

The authors did not receive any financial support for this study.

## CONFLICT OF INTEREST STATEMENT

The authors have no conflict of interest to declare.

## Supporting information


**Table S1.** Study list of procedures and approaches for the 3‐month training period.

**Table S2.** Objective Structured Assessment of Technical Skills (OSATS) global rating scale (GRS).
This material is available as part of the online article from: *(electronic link to be added after preparation of galley proofs)*.

## Data Availability

The datasets used and analyzed during the current study are available from the corresponding author on reasonable request.
