# Supplementary material for: Structured assessment of a cadaveric orthopedic surgical training program of small animal surgeons in training: A prospective observational pilot study
Source: Vet Surg. 2025 Oct 31;55(3):597–604. doi: 10.1111/vsu.70033 (PMC13069220; doi:10.1111/vsu.70033)
Supplement: Supplementary file 1 — Table S1. Study list of procedures and approaches for the 3‐month training period. [file VSU-55-597-s002.docx]

**Table S1. List of procedures and approaches for the 3-month training period**

| General principles^a,b^ |  | Skin cut |
| --- | --- | --- |
|  |  | Blunt dissection |
|  |  | Appropriate use of retraction |
|  |  | Use of instruments |
|  |  | Tissue handling |
|  |  | Suturing / Hand tie |
|  |  | Efficacy |
|  |  | Retraction and surgical exposure |
|  |  | Use of assistants |
| Approach to long bones^c^ | Humerus | Approach to the proximal shaft of the humerus (and collect bone graft) |
|  |  | Approach to the midshaft of the humerus through a craniolateral incision |
|  |  | Approach to the shaft of the humerus through a medial incision |
|  |  | Approach to the distal shaft of the humerus through a craniolateral incision |
|  | Radius/Ulna | Approach to the head and proximal metaphysis of the radius |
|  |  | Approach to the shaft of the radius through a medial incision |
|  |  | Approach to the shaft of the radius through a lateral incision |
|  |  | Approach to the distal radius and carpus through a dorsal incision |
|  | Femur | Approach to the shaft of the femur (lateral) |
|  |  | Approach to the distal femur and stifle joint through lateral incision |
|  | Tibia | Approach to the proximal tibia through a medial incision |
|  |  | Approach to the shaft of the tibia (medial) |
|  | Pelvis | Approach to the wing of the ilium and dorsal aspect of the sacrum (and collect bone graft) |
|  |  | Approach to the ilium through a lateral incision (“gluteal roll-up”) |
|  |  | Approach to the ilium through a lateral incision (“gluteal roll-down”) |
|  |  | Approach to the ischium |
| Approach to joints^c^ | Elbow joint | Approach to the head of the radius and lateral part of the elbow joint |
|  |  | Approach to the lateral humeroulnar part of the elbow joint |
|  |  | Approach to the medial aspect of the humeral condyle and the medial coronoid process of  the ulna by an intermuscular incision |
|  | Carpal joint | Approach to the distal radius and carpus through a dorsal incision |
|  |  | Approach to the distal radius and carpus through a palmaromedial incision |
|  | Hip joint | Approach to the craniodorsal aspect of the hip joint through a craniolateral incision |
|  |  | Approach to the dorsal aspect of the hip joint through an intergluteal incision |
|  |  | Approach to the caudal aspect of the hip joint and body of the ischium  Approach to the craniodorsal and caudodorsal aspects of the hip joint by osteotomy of the  greater trochanter |
|  | Stifle joint | Approach to the stifle joint through a lateral incision |
|  |  | Approach to the stifle joint through a medial incision |
|  |  | Approach to the stifle joint with bilateral exposure |
|  | Talocrural joint | Approach to the lateral malleolus and talocrural joint |
|  |  | Approach to the medial malleolus and talocrural joint |
| Specific procedures^a,b^ |  | Placing an intramedullary pin (femur, tibia) |
|  |  | Insert parallel pins |
|  |  | Create an oblique fracture with an oscillating saw |
|  |  | Apply a screw in lag or neutral fashion for an oblique fracture |
|  |  | Apply a cerclage wire for an oblique fracture |
|  |  | Fixation of trochanter osteotomy with cerclage compression wiring |

^a^ Reference: Johnston SA, Tobias KM. *Veterinary Surgery: Small Animal*. 2nd ed. Elsevier Saunders; 2017.
^b^ Reference: DeCamp CE, Johnston SA, Déjardin LM, Schaefer SL.*Brinker,* *Piermattei and Flo’s Handbook of Small Animal Orthopedics and Fracture Repair*. 5th ed. Elsevier Saunders; 2016.
^c^ Reference: Johnson KA. *Piermattei's Atlas of Surgical Approaches of the Bones and Joints of the Dog and Cat*. 5th ed. Elsevier Saunders; 2014.
