# Supplementary material for: Structured assessment of a cadaveric orthopedic surgical training program of small animal surgeons in training: A prospective observational pilot study
Source: Vet Surg. 2025 Oct 31;55(3):597–604. doi: 10.1111/vsu.70033 (PMC13069220; doi:10.1111/vsu.70033)
Supplement: Supplementary file 2 — Table S2. Objective Structured Assessment of Technical Skills (OSATS) global rating scale (GRS). This material is available as part of the online article from: (electronic link to be added after preparation of galley proofs). [file VSU-55-597-s001.docx]

| **1) Respect for tissue** |  |  |  |  |  |
| --- | --- | --- | --- | --- | --- |
| **1** | **2** | **3** | **4** | **5** | **Score** |
| Frequently used unnecessary force on tissue or caused damage by inappropriate use of instruments |  | Careful handling of tissue but occasionally caused inadvertent damage |  | Consistently handled tissues appropriately with minimal damage |  |
| **2) Time and Motion** |  |  |  |  |  |
| **1** | **2** | **3** | **4** | **5** | **Score** |
| Many unnecessary moves |  | Efficient time/motion but some unnecessary moves |  | Clear economy of movement and maximum efficiency |  |
| **3) Instrument Handling** |  |  |  |  |  |
| **1** | **2** | **3** | **4** | **5** | **Score** |
| Repeatedly makes tentative or awkward moves with instruments |  | Competent use of instruments but occasionally appeared stiff or awkward |  | Fluid moves with instruments and no awkwardness |  |
| **4) Knowledge of Instruments** |  |  |  |  |  |
| **1** | **2** | **3** | **4** | **5** | **Score** |
| Frequently used an inappropriate instrument |  | Used appropriate instruments for the task |  | Obviously familiar with the instruments required and their use |  |
| **5) Use of proper retraction *** |  |  |  |  |  |
| **1** | **2** | **3** | **4** | **5** | **Score** |
| Consistently placed retractors poorly or failed to use retractors |  | Appropriate use of retractors most of the time |  | Strategically used retractors to the best advantage at all times |  |
| **6) Flow of Operation** |  |  |  |  |  |
| **1** | **2** | **3** | **4** | **5** | **Score** |
| Frequently stopped operating and seemed unsure of next move |  | Demonstrated ability for forward planning with steady progression of operative procedure |  | Obviously planned course of operation with effortless flow from one move to the next |  |
| **7) Knowledge of specific procedure** |  |  |  |  |  |
| **1** | **2** | **3** | **4** | **5** | **Score** |
| Deficient knowledge. Needed specific instruction at most operative steps |  | Knew all important steps of the operation |  | Demonstrated familiarity with all aspects of the operation |  |
|  |  |  |  | **Total score:** |  |

**Table S2. Objective Structured Assessment of Technical Skills (OSATS) global rating scale (GRS)**
Number of video/name of study participant:
Name of supervising surgeon:
Please score corresponding to the candidate’s performance in each category

* Modified from the original OSATS (“Use of assistants” was replaced by “Use of proper retraction”)
